# Supervised Patient Self-Testing of Warfarin Therapy Using an Online System

**DOI:** 10.2196/jmir.2255

**Published:** 2013-07-12

**Authors:** Luke Ryan Elliot Bereznicki, Shane Leigh Jackson, Gregory Mark Peterson

**Affiliations:** ^1^Unit for Medication Outcomes Research and EducationSchool of PharmacyUniversity of TasmaniaHobartAustralia

**Keywords:** warfarin, self-care, management, international normalized ratio, Internet, communication

## Abstract

**Background:**

Point-of-care international normalized ratio (INR) monitoring devices simplify warfarin management by allowing selected patients to monitor their own therapy in their homes. Patient self-testing (PST) has been shown to improve the clinical outcomes of warfarin therapy compared to usual care.

**Objective:**

To compare management of warfarin therapy using PST combined with online supervision by physicians via a custom system with usual warfarin management, which involved laboratory testing and physician dosing.

**Methods:**

Interested patients were recruited via community pharmacies to participate in a warfarin PST training program. Participants were required to have a long-term indication for warfarin, have been taking warfarin for at least 6 months, and have Internet access in their home. The training involved two sessions covering theoretical aspects of warfarin therapy, use of the CoaguChek XS, and the study website. Following training, patients monitored their INR once weekly for up to 3 months. Patients and physicians utilized a secure website to communicate INR values, dosage recommendations, and clinical incidents. Physicians provided a 6-12 month history of INR results for comparison with study results. The percentage of time spent within the therapeutic INR range (TTR) was the primary outcome, with participants acting as their own historical controls. The percentage of INR tests in range and participant satisfaction were secondary outcomes.

**Results:**

Sixteen patients completed training requirements. The mean age of participants was 69.8 (SD 10.1) years. TTR improved significantly from 66.4% to 78.4% during PST (*P*=.01), and the number of tests within the target range also improved significantly (from 66.0% at prior to the study to 75.9% during PST; *P*=.04). Patients and physicians expressed a high degree of satisfaction with the monitoring strategy and online system.

**Conclusions:**

PST supported by an online system for supervision was associated with improved INR control compared to usual care in a small group of elderly patients. Further research is warranted to investigate the clinical outcomes and cost-effectiveness of online systems to support patients monitoring medications and chronic conditions in the home.

## Introduction

Warfarin is an anticoagulant that has been, and continues to be, the standard of care to prevent and treat thromboemboli. It is estimated that between 1% and 2% of the population of the developed world currently receives oral anticoagulants, predominantly warfarin, on a regular basis, mainly for the prevention of ischemic stroke associated with chronic atrial fibrillation. While newer anticoagulants have become available for some indications, their high cost, limited range of indications, and uncertain risk/benefit profile, particularly in the elderly, ensure that debate continues regarding their place in therapy as either replacements or alternatives to warfarin [1-3]. While effective, warfarin is a well-known cause of adverse drug events, and it is fundamental that efforts are made to improve the safety of warfarin therapy and maximize its clinical benefits.

Despite decades of clinical use, there are a number of aspects of warfarin therapy that can be targeted to improve patient outcomes. Initial dosing can be optimized through the application of dosing algorithms based on clinical and genetic parameters [4], and patient knowledge of warfarin (often described as suboptimal in the literature) can be improved through health professional intervention [5,6]. Dietary vitamin K intake can be optimized in some patients to improve control [7], and the use of interacting medications can be minimized through judicious prescribing. While the need for regular monitoring of the international normalized ratio (INR), necessitated by large interindividual differences in response to warfarin, can be a burden to patients and health care systems, the ready availability of INR testing means that warfarin therapy can be rigorously monitored to optimize patient safety. The degree of INR control is the major determinant of the efficacy and safety of warfarin therapy [8], and optimized monitoring may make warfarin more clinically effective and cost-effective than its competitors [9,10]. Of course, if warfarin therapy is not well controlled, patients are at increased risk of thrombotic and hemorrhagic complications [11].

Traditionally, warfarin is managed by primary care physicians, pathology providers, or by health professionals in dedicated anticoagulation clinics. Point-of-care (POC) devices offer an accurate alternative to laboratory monitoring [12,13], and their availability has led to an increased focus on patient self-testing (PST) of warfarin. This may comprise self-testing, where the result of the test is communicated to a physician for management (usually by phone), or self-management, where the patient self-adjusts the warfarin dose. PST has been evaluated in a number of well-controlled studies, and a recent meta-analysis demonstrated that self-monitoring of warfarin therapy results in significant reductions in the incidence of thromboembolic complications [14]. The reasons that PST can be more effective than usual care are multifactorial and include enabling an increase in testing frequency, educating patients about important aspects of warfarin therapy, and empowering patients to take a greater role in their own care.

It is possible that the appropriate application of information technology could greatly improve warfarin management by improving communication between patients and physicians and facilitating self-monitoring. The Internet offers significant promise as an enabling factor for PST by allowing patients to monitor their therapy at home and receive ongoing advice without having to visit their supervising health professional, particularly in situations where patients and/or their supervisors may be uncomfortable with the patient taking unsupervised control of their condition.

We sought to develop and test an approach to improve INR control by training patients to self-test their INR and linking them to their primary care physician with an online support system. Our hypothesis was that self-testing with online support would improve INR control compared to usual care. This required the development of a training process to enable participants to self-test and an online system to link patients with their physicians and support staff. We conducted a prospective, proof-of-concept study to compare the INR control achieved with online-supervised PST to usual care, investigate patient and physician views on the online model of care, and provide the foundation for more extensive research in this area.

## Methods

### Design

This was a prospective study of PST with online decision support. Participants acted as their own historical controls. The INR control achieved with PST was compared with the INR control achieved in the 6-12 months immediately preceding the study using conventional management (laboratory INR with physician dose adjustment).

### Patient Recruitment

To participate in this study, patients had to have been taking warfarin for at least 6 months, have a long-term indication for warfarin therapy, be willing to participate in training to enable PST, and have Internet access in the home. To gauge patient interest in PST, we randomly selected 20 community pharmacies in southern Tasmania to participate in a preliminary survey (using random number generation and a list of pharmacies). These pharmacies contacted all patients who had been dispensed warfarin in the past 3 months and mailed them an information sheet, which invited patients to contact the researchers if they were interested in participating in a survey regarding their interest in PST. A follow-up survey of patients who indicated a willingness to be contacted to provide additional information was conducted to identify patients who met the inclusion criteria. These patients were approached to provide their consent to be involved in the study. Once consent was provided, the research team contacted the patient’s primary care physician informing them of the study and their patient’s interest, and seeking their consent to participate. Physician consent was essential for the patient to be included in the trial. Ethics approval was provided by the Southern Tasmania Health and Medical Human Research Ethics Committee.

### Patient Training

Once enrolled in the trial, patients were trained to use a portable INR monitor (CoaguChek XS, Roche Diagnostics Australia) by the research team. The education consisted of two sessions of 1-2 hours duration held approximately 1 week apart. The first session covered background information on warfarin, risk of bleeding, diet, the INR, and a practical demonstration of the CoaguChek XS, and was conducted as a small group session (2-6 patients). Patients then received their own monitor and were asked to conduct 4-5 tests prior to the next session to become accustomed to the device and confident in their testing technique.

A “run-in” phase, where patients completed 2 INR tests on the CoaguChek XS in conjunction with 2 pathology tests to compare for accuracy, ensured that the research team, the physician, and the patient were satisfied that the monitor provided accurate results and could be used effectively. Patients were asked to complete these comparison tests prior to the second education session. If the CoaguChek INR results were not suitably accurate (defined as the CoaguChek XS INR being within 15% of the laboratory INR) on both occasions, further instruction was provided. If further comparisons were not suitably accurate, the patient was excluded from the trial. The second session covered other aspects of using the monitor, such as quality control and using the online system to relay the results to the physician. This session was conducted as a home visit by one of the researchers to ensure effective use of the patient’s home computer. The physicians also received some instruction on how to access and utilize the online system. This education was delivered at the physician’s surgery in a one-on-one session with a member of the research team.

An observed assessment was made of each participant before home monitoring could occur. Patients were required to demonstrate that they could use the CoaguChek XS in a proficient manner and satisfactorily complete a test based on the theory content of the course.

### Website Development and Functionality

A consultant information technology company, in collaboration with the research team, developed the online system used in the study. In order to tailor the website to the needs of the end users, the research team conducted two focus group meetings, with patients and physicians. The facilitated discussions were aimed at improving the design of the website and identifying, in particular, which functions were required, flow of information, and training requirements.

The self-testing and data entry procedure consisted of performance of the INR test on the POC INR monitor, logging on to the secure website, and manual entry of the test result. The system provided an overview of the steps required in the testing process and asked users to indicate if they had conducted the test appropriately. Following entry of the result by the patient, the system displayed the result and asked the patient to confirm its accuracy. The system screened the result and noted whether the test was below, within, or above the prescribed INR range and asked the patient to document any changes in diet, medication, or make any additional comments. Finally, the system requested confirmation of all information entered and sent an alert to the physician via email.

Physicians were asked to respond to the test result within 24 hours and provide the warfarin dose and date of the next test. Physicians were free to log on to the secure website and view patient details at any stage. Physicians were able to make a recommendation based on the INR, information provided by the patient, and stored history. The system notified the patient of the dosage recommendation when it was available from the physician via email. The system was used by physicians to set the date of the next test and alerted support staff if tests became overdue. If a patient had not completed an INR test within 24-48 hours of the test being due, the researchers contacted the patient. Similarly, the physician was to be contacted if the patient had not received a dosage recommendation within 24-48 hours of completing the INR test. Telephone support was available from the research team at all times.

The online system allowed patients to access to a variety of educational materials related to anticoagulation, and dietary and lifestyle advice for patients taking warfarin. Patients were also able to visualize their INR results on a graph and view physician recommendations. The system stored patient information, including contact details and INR history; access to this information by patients and physicians was only available through a password-protected website. Patients had access to only their own details while physicians were able to access information relevant to all patients under their care. Support staff had access to all information. Communications to and from this site were encrypted. Emails did not contain any sensitive information. All data were physically stored in a secure environment and treated confidentially and anonymously. For screenshots of the online system, see Multimedia Appendix 1.

### International Normalized Ratio Testing

Once training was complete, participants tested their INR approximately once per week, or more or less frequently if required by the physician, for a period of up to 3 months. The percentage of time in therapeutic range (TTR) and percentage of tests within the INR target range were determined for each patient during the trial. This was compared to their previous level of control for the 6-12 months immediately prior to the commencement of the PST phase of the study (provided at study entry with each participant’s consent). The function to calculate the TTR was based on the method of linear interpolation originally proposed by Rosendaal et al [15].

### Sample Size and Statistical Analysis

The primary outcome was change in TTR from baseline. The TTR for each participant was determined for prePST and PST data. Paired *t* tests were used to determine if any significant change had occurred compared to baseline. Statistical significance was set at *P*<.05. Feedback from physicians and patients was sought regarding the system and its ease of use. This was obtained using evaluation questionnaires featuring visual analogue scales, ranging from 0 to 10. Scores <5 indicated disagreement with the statements provided, while scores >5 indicated agreement with the statements provided. The study was not powered to detect statistical differences in clinical outcomes, such as bleeds, although their occurrence was documented as a matter of course. All information was stored and analyzed using SPSS 19.0 (version 19.0 for Mac).

A sample size of approximately 20 patients was deemed be adequate to demonstrate the feasibility of this type of warfarin management. The literature suggests that patients in the community spend 50-60% of their time within the target range [16]. It was envisaged that this could be improved to 75% with the intervention based on a prior study involving a similar intervention [17]. At a power of 80% and statistical significance set at 0.05, 16 patients analyzed before and after were required.

## Results

### Participants

**Figure 1:**
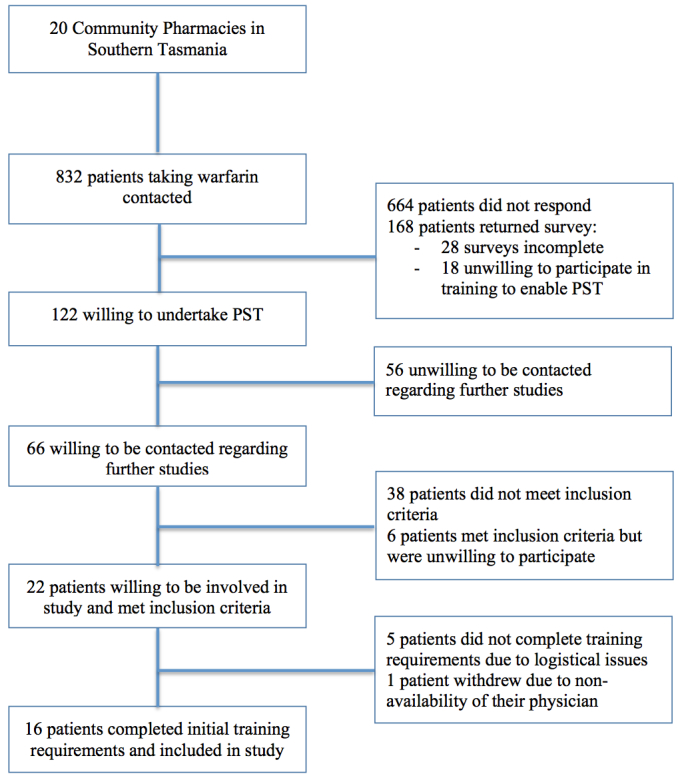
Patient recruitment.

Figure 1 shows a summary of the recruitment process. Of the 832 patients contacted by their community pharmacies, 243 (29.2%) contacted the researchers. One hundred and sixty-eight patients returned the survey (69.1%), of whom 122 (72.6%) indicated a willingness to undertake training to enable PST. A follow-up survey of 66 of these patients who indicated a willingness to be contacted to provide additional information identified 28 potential participants in the study who reported having Internet access in their home and met the other inclusion criteria. Twenty-two of the 28 patients (78.6%) who met the inclusion criteria consented to participate in the trial. Five patients did not complete the training requirements due to logistical issues, and 1 patient did complete some of the training requirements but was unable to continue in the study due to the unavailability of their physician. Sixteen patients completed the initial training program and went on to perform PST. Sixteen different physicians were involved in the management of these patients. Once training was complete, there were no withdrawals from the study. Table 1 displays patient characteristics for the patients who performed PST.

### Device Accuracy

A total of 59 comparison INRs (CoaguChek XS and laboratory INR conducted within 4 hours of each other) were completed either on entry into, or during the trial by the participants. The CoaguChek XS INR values were significantly correlated with the laboratory INR values (*r*=0.91, *P*=.01). The mean difference in INR (laboratory minus CoaguChek XS) was 0.07 (SD 0.06) (*t*_58_=2.56, *P*=.01).

### International Normalized Ratio Control

**Figure 2:**
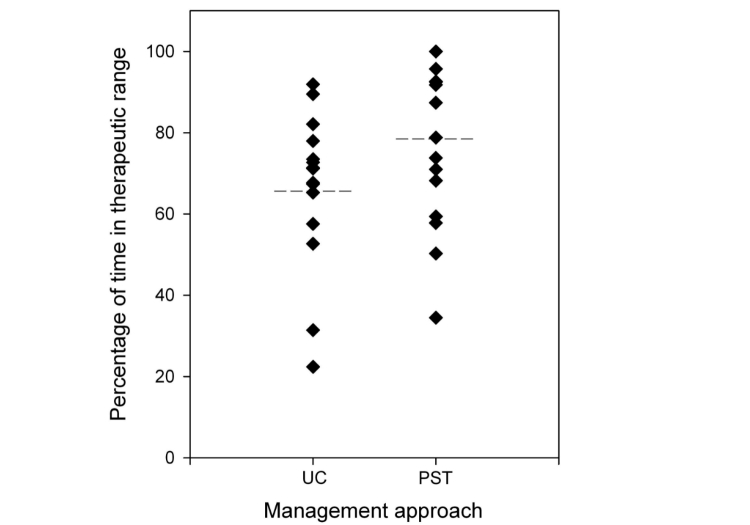
Individual percentage of time in therapeutic range (TTR) during patient self-testing (PST) and usual care (UC) (the dotted line shows the mean TTR for each management approach).

**Figure 3:**
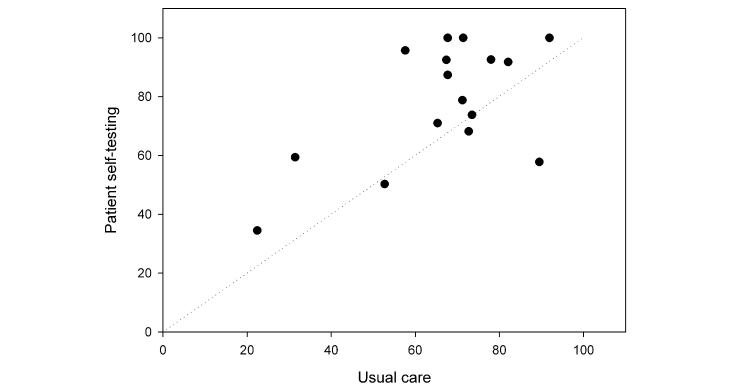
Percentage of time in therapeutic range for patient self-testing vs usual care.

The mean TTR prior to PST was 66.4%(SD 17.7%). A total of 309 INR tests were provided with an average of 19.3 (SD 7.9) tests per patient. The mean duration of time encompassed by the baseline data was 338.4 (SD 52.8) days. During PST, patients had a mean TTR of 78.4% (SD 20.1%). The mean number of results per patient was 7.5 (SD 3.0; 120 home tests were completed by the cohort. The mean duration of PST was 45.1 (SD 16.0) days. Figure 2 shows a comparison of mean TTR during usual care and PST. There was a statistically significant improvement in the TTR when patients performed PST (mean improvement 12.0% (SD 17.3%), *P*=.01) (Table 2). The percentage of time spent below and above target range were reduced nonsignificantly. Thirteen of 16 (81.3%) patients improved on their baseline control (Figure 3). The mean increase in the number of tests in target range was 9.9% (66.0%, SD 16.6% usual care, 75.9%, SD 19.2% during intervention, *P*=.04), a significant improvement. No clinical outcomes (events of major bleeding or thromboembolism) were observed during the intervention.

### Participant Evaluation

At the completion of the trial, questionnaires were sent to all physicians and patients. The response rate for physicians was 87.5% (14/16) and for patients was 93.8% (15/16).

**Table 1:** Patient characteristics.

Characteristic		n (%)
Male sex		12 (75.0)
Mean age (SD years)		69.8 (10.1)
**Indication for warfarin**		
	Atrial fibrillation	7 (43.8)
	Venous thrombosis	2 (12.5)
	Heart valve	6 (37.5)
	Other	1 (6.3)
**Target INR range**		
	2.0-3.0	10 (62.5)
	2.5-3.5	2 (12.5)
	Other (specified by physician)	4 (25.0)

**Table 2:** Percentage of time in target range.

	Usual care	Patient self-testing	Mean change	*P*
% within range (SD)	66.4 (17.7)	78.4 (20.1)	+12.0 (17.3)	.01
% below range (SD)	22.2 (24.3)	13.5 (15.5)	-8.7 (20.2)	.11
% above range (SD)	11.4 (10.2)	8.1 (11.1)	-3.2 (14.0)	.37

### Physician Evaluation

Physicians indicated that they found the intervention to be a beneficial service for their patients (median score 7.5, range 6.2-8.9). They were also positive when asked whether they would feel more confident in managing patients taking warfarin if it was a regular service (median score 7.0, range 3.0-8.3). Physicians strongly agreed that they were confident in the accuracy of POC INR results (median score 7.5, range 5.0-9.3) and tended to agree that the system was easy to use (median score 7.0, range 4.7-9.5). Physicians generally found that receiving, reviewing, and responding to an INR result took 1-3 minutes. Most physicians responded positively (median score of 8.0, range 2.6-9.5) when asked if they saw this model of care as a feasible way to manage patients taking warfarin in the future, and believed that more patients would benefit from this service (median score 7.5, range 5.0-8.4).

### Patient Evaluation

All patients who responded to the evaluation questionnaire found the intervention to be a worthwhile service (median score 9.5, range 5.5-9.5) and would feel more confident about taking warfarin if it was offered as a regular service (median score 7.0, range 5.5-9.5). Most patients indicated they preferred self-testing compared to laboratory INR testing (median score 7.5, range 4.5-9.5). Patients felt strongly that the education and training provided was of benefit to them (median score 9.5, range 6.9-9.5) and generally felt that their participation had improved their knowledge regarding their treatment (median score 7.5, range 5.2-9.5). They felt that the portable monitor was easy to use (median score 7.5, range 5.5-9.5) and were confident in its accuracy (median score 9.3, range 5.7-9.5). They also reported that the website was easy to use (median score 7.5, range 5.7-9.5) and were satisfied with the support provided (median score 9.5, range 7.5-9.5). They reported that they generally spent between 1 and 3 minutes entering INR results on the system or checking the dose changes provided by their physicians.

## Discussion

### International Normalized Ratio Control

This study found that INR control improved when patients performed PST and were remotely supervised by their physicians using a custom online system, compared to their usual care under the same physician in a small number of patients. Participants received training and used the CoaguChek XS to monitor their INR, entered their INR into an online system, and received advice from their supervising physician. Patients and physicians alike found it to be a valuable model of care for warfarin therapy.

There is a direct relationship between TTR and clinical outcomes for patients taking warfarin [11,18]. The generalizability of the results of international trials comparing new anticoagulants to warfarin depends largely on the TTR achieved in the trials and the TTR achieved by patients taking warfarin within the particular health care systems studied. It has been established that the relative efficacy and safety of comparator drugs to warfarin varies depending on the quality of INR control [9]. Therefore, the TTR is critical in determining the relative efficacy, safety, and potential cost-effectiveness of new anticoagulants compared to warfarin, as well as comparing various models of warfarin management. We found that TTR improved from 66% to 78% with the model of care developed in this study. The usual care mean TTR of 66% was higher than we expected; a mean TTR in the order of 50-60% was anticipated based on previous community-based nonrandomized studies [16]. Interestingly, the usual care mean TTR was higher than that achieved in several recent randomized trials comparing warfarin to new anticoagulants, even though the TTR in the trials is often said to be higher than that achieved in practice [19-21]. This is possibly due to the recruitment process, which identified patients who were interested in PST and were perhaps more motivated and knowledgeable regarding their treatment than other people taking warfarin. However, it is notable that even in a group with relatively good baseline INR control, it was possible to achieve a TTR approaching 80% with PST. Improvements in TTR of this magnitude are likely to be associated with improved clinical outcomes. A retrospective study in patients with atrial fibrillation found that a 7% improvement in TTR is associated with 1 fewer hemorrhagic event per 100 patient-years and a 12% improvement is associated with 1 fewer thromboembolic event per 100 patient-years [22].

Self-monitoring often results in improved INR control compared to the control achieved in comparator groups (either primary care or anticoagulation clinic management) [14]. A number of studies have shown improved TTR [23-25], while in other studies PST has not resulted in an increase in TTR but measures of stability have improved [26,27]. TTR may not be the only means by which PST might result in improved clinical outcomes. In a trial by Menedez et al [26], TTR did not improve significantly between the usual care group and the self-managing group but the incidence of major warfarin-related complications was reduced significantly. The beneficial effects of PST may therefore relate to patient education [6], patient empowerment [28,29], or improved medication adherence [30], in addition to improving measures of INR control.

The INR testing frequency in this study was weekly unless otherwise specified by the supervising physician. In the prePST phase, the mean testing frequency was approximately 18 days. Some researchers argue that the improvements in TTR generally associated with PST are largely due to an increased testing frequency [31-33]. This may not necessarily be the case as PST has been shown to improve the TTR without a change in testing frequency [34], and another study recently found that a longer testing interval (12 weeks) was not associated with any change in mean TTR compared to a 4-week testing interval [35]. In fact, more frequent testing may actually have a detrimental effect on TTR, as it may lead to unnecessary dose adjustment [35]. Regardless, our study was not designed to differentiate between the potential individual benefits of patient education, increased testing frequency, PST, or the presence of the online system.

PST provides the flexibility to monitor more or less frequently at the discretion of the physician without creating undue pressure on the patient to attend pathology testing and/or physician consultations. A weekly testing frequency can also be achieved with PST at a similar cost to monthly laboratory testing. In a Canadian study, the ongoing costs associated with weekly self-testing were identical to the costs of 4-weekly conventional monitoring [36].

### Comparison With Prior Work

Our results are consistent with the improvements in TTR associated with PST and online systems in three other studies [17,37,38]. The studies by O’Shea et al [17] and Ryan et al [37] used the same Internet-based system, which provided a decision-support tool to assist patients adjust their own warfarin dose. The improvements in INR control achieved in each of these studies were similar. The TTR improved from 63% to 74% (*P*<.01) in the pilot investigation [17] and from 59% to 74% (*P*<.01) in the subsequent randomized controlled trial [37]. The study by Harper and Pollock used a different online system to support PST [38]. This system provided instant feedback on the warfarin dose if the INR was in the therapeutic range, but if outside the range sent an alert to the physician to review. The TTR improved nonsignificantly from 72% to 81%. Unlike in these studies, the system used in our study was not designed to provide any dosing advice to patients—in all instances, the test result was sent to physicians for review. It is interesting that this difference did not appear to affect the TTR achieved with PST and the online system, although a larger comparative study would be required to verify this observation.

Our study is noteworthy for two other reasons. First, our patients were older than those studied in previous studies involving online systems. The mean age of our participants was 70 years, while the median age was 54 years in the study by O’Shea [17] and the mean age was 59 years in the larger study by Ryan [37]. Our patients were also older than those in almost all previous studies of PST [14]. This is important because warfarin is increasingly indicated in an older population, but advanced age is often seen as a deterrent to warfarin therapy because of a perceived increase in the risk of bleeding [39]. Our results suggest that not only can excellent INR control be achieved in older patients, they can also be trained to successfully perform PST and use online systems. Our participants were a selected population; nonetheless, the results indicate that advanced age should not necessarily be considered a deterrent to achieving excellent INR control, utilizing PST or online systems. Clearly, these observations need to be tested in larger, long-term studies.

Second, we have provided data on the experience of our end users, both physicians and patients. Our online system was designed with input from end users—this led to a focus on ease of use, convenience, and safety based on their priorities. The physicians who were involved in the pilot study and completed an evaluation questionnaire all found it to be a valuable service for their patients. Physicians agreed that more patients would benefit from this type of service and the percentage of patients that they felt this system would be suitable for ranged from 12% to 98%. Our experience in this study suggests that most of these patients would be capable of completing the necessary training to self-test and use the online system. Previous research in the United Kingdom using an unselected population gave similar results in terms of capability to self-test [40]. As far as using the system was concerned, physicians responded that the system was easy to use and the warfarin home monitoring website was easy to navigate. The majority of physicians did not require any additional support following their initial training.

Importantly, the patients using the online system found it to be a valuable service that made them feel more confident about their warfarin therapy. They found the initial training beneficial and also agreed that their warfarin knowledge had improved as a result of the training. Importantly for the ongoing development of the system, patients found the website training easy and were highly satisfied with the ongoing support and by their physicians’ involvement and use of the system. Patients reported spending the same amount of time on the warfarin home monitoring trial website as their physicians, that is, 1-3 minutes per test. When asked whether they would prefer to monitor their warfarin therapy at home, patients indicated that, in general, they would.

### Limitations

Our study involved a selected group of participants who may not be representative of the broader population of people taking warfarin. It is possible that they were more motivated and possibly more adherent with their therapy than other patients [33]. Other potential limitations of the study include the small sample size, nonrandomized design, and relatively short duration of the intervention.

### Conclusions

This proof-of-concept study was successful in demonstrating the feasibility of an alternative warfarin management strategy involving supervised PST using an online system in a small group of selected participants. Patients spent more than 78% of time in the therapeutic range while self-testing, which was a significant improvement from their previous INR control. Patients and physicians were highly satisfied with the monitoring system. Further research is warranted to investigate the benefits and implications of this strategy for people taking warfarin, as well as other narrow-therapeutic index drugs and those with chronic diseases where regular monitoring is indicated.

## Abbreviations

INR: international normalized ratio
POC: point-of-care
PST: patient self-testing
TTR: time in therapeutic range
UC: usual care

## Multimedia Appendix 1

Screenshots of the Web-based system.
