# Supplementary material for: Supervised Patient Self-Testing of Warfarin Therapy Using an Online System
Source: J Med Internet Res. 2013 Jul 12;15(7):e138. doi: 10.2196/jmir.2255 (PMC3713938; doi:10.2196/jmir.2255)
Supplement: Supplementary file 1 [file jmir_v15i7e138_app1.pdf]

Warfarin Home Monitor Trial

Please follow the testing procedure below:

1
2
3
4
5
6

Follow Coaguchek Testing Procedure

1. Ensure that the device is on a stable, flat surface.
2. Check that the code displayed matches the last three digits on the test strip foil pouch.
3. Check that the time and date are correctly set.
4. Remove test strip from pouch and insert into the device.
5. Wait until the device displays the countdown from 180 - ensure that the sample is applied to the test strip before the countdown reaches zero.
6. Prick the tip of your middle or ring finger at the side using the lancet device.
7. Rub the outside of the finger until a large, hanging drop of blood has formed - DO NOT PINCH OR SQUEEZE.
8. Apply the drop of blood to the flashing yellow area of the test strip within 15 seconds of pricking your finger.
9. Check that the INR reading is displayed after the clock icon disappears.

Next

Instructions

If a problem is encountered during this procedure, please consult the quick reference guide included with the device. If you need further help, please send an email to Dr. Alice Lynch at alynch@dr.com or call 0411 888 4444

Warfarin Home Monitor Trial

HOME (ALERTS) | HISTORY | SCHEDULE | INFO | CONTACT GP | SUPPORT | LOGOUT

Please follow the testing procedure below:

1
2
3
4
5
6

Enter INR Test Result For Review

1. Check that the test result is shown on Coaguchek display.
2. Enter the INR result below:

INR : 1.5

Send Result

Warfarin Home Monitor Trial

HOME (ALERTS) | HISTORY | SCHEDULE | INFO | CONTACT GP | SUPPORT | LOGOUT

Please follow the testing procedure below:

1
2
3
4
5
6

Confirm test result

You have entered a test result of 1.5 (INR)

1.5 INR

Enter Again

Continue

Instructions

Check that this number is the same as the one displayed on the device screen.
If this is not correct, please click on the "Change Result" button; otherwise, please click on the "Next" button.

Warfarin Home Monitor Trial

HOME (ALERTS) | HISTORY | SCHEDULE | INFO | CONTACT GP | SUPPORT | LOGOUT

Please follow the testing procedure below:

1
2
3
4
5

IMPORTANT

Test result is Outside Prescribed Range

1.5 INR

The test result of 1.5 (INR) is outside the prescribed range of 1.7 -2.7.

Please add a comment

Previous

Continue

Please add a comment on any circumstances which may have resulted in an unusual test result. This comment will assist your GP in reviewing this reslt.
Please include details on :

- Any new mediation
- Any recent medical care which may not be known to Dr..........
- Any dietary changes

Warfarin Home Monitor Trial

Please follow the testing procedure below:

1
2
3
4
5

Send Test Results for Review

1.5 INR

Please click on either of the buttons below to send the results to your GP for review.
Your GP will review your results and send a response whitn 24 hours.

Send Results and View Your History

Send Results and Return to Main Menu

Warfarin Home Monitor Trial

HOME (ALERTS) | HISTORY | SCHEDULE | INFO | CONTACT GP | SUPPORT | LOGOUT

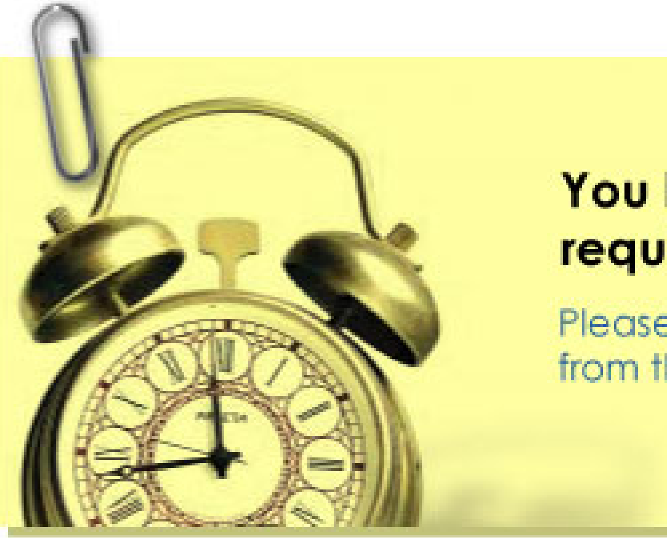

You have 1 pending alerts requiring atteniton.

Please resolve them by selecting from the list below.

ALERTS REQUIRING ATTENTION

Date & Time		Description	Status
29 Aug 2005	14:09	Review of INR Test Results<br>click to view results	Ready
28 Aug 2005	9:00	Home INR Test<br>Waiting for home INR test results.<br>click to take test	Overdue
27 Aug 2005	9:00	Home INR Test<br>Waiting for home INR test results.<br>click to take test	Escalated

MAIN MENU:

- VIEW SCHEDULE
- VIEW PATIENT RECORDS
- ENROL NEW PATIENT
- VIEW TRAINING INFORMATION
- CONTACT SUPPORT
- LOGOUT
